# Challenges in defining the rates of ADHD diagnosis and treatment: trends over the last decade

**DOI:** 10.1186/s12887-017-0971-0

**Published:** 2017-12-29

**Authors:** Michael Davidovitch, Gideon Koren, Naama Fund, Maayan Shrem, Avi Porath

**Affiliations:** 1 Department of Child Development, Medical Division, Maccabi Healthcare Services, 27 Hamered St., 6812509 Tel Aviv, Israel; 2 Research Institute, Maccabi Healthcare Services, Tel Aviv, Israel; 3 Western University, Ontario, Canada; 4 Department of Health Services Research, Maccabi Healthcare Services, Tel Aviv, Israel; 5 Faculty of Medicine in the Galilee, Bar-Ilan University, Safed, Israel; 6 Chief Physician Office, Medical Division, Maccabi Healthcare Services, Tel Aviv, Israel; 7 Department of Epidemiology, Ben-Gurion University of the Negev, Beer Sheva, Israel

**Keywords:** ADHD, Prevalence, Incidence, Treatment

## Abstract

**Background:**

There is a global trend of large increases in the prevalence and incidence of Attention Deficit Hyperactivity Disorder (ADHD). This study aimed to address potential causes of these major changes.

**Methods:**

The authors used a large cohort to analyze data employing patients’ electronic medical records, with physicians’ diagnosis of ADHD, including records of medication purchases.

**Results:**

The prevalence of ADHD diagnoses rose twofold from 6.8% to 14.4% between 2005 and 2014 (*p* < 0.001), while the ratio of males to females with ADHD decreased from 2.94 in 2005 to 1.86 in 2014 (*p* < 0.001). The incidence increased, peaking in 2011 before declining in 2014. ADHD medication usage by children and adolescents was 3.57% in 2005 and 8.51% by 2014 (*p* < 0.001).

**Conclusions:**

We report a dramatic increase in the rate of ADHD diagnoses. One of the leading factors to which we attribute this increase is the physicians’ and parents’ changed attitude towards diagnosing attention/hyperactivity problems, with more parents appear to consider ADHD diagnosis and treatment as a means to improve their child’s academic achievements, commonly with the aid of medications. This change in attitude may also be associated with the dramatic increase in female ADHD diagnosis prevalence.

## Background

Attention Deficit Hyperactivity Disorder (ADHD) is one of the more common neurobehavioral disorders in the world, making its diagnosis and treatment an area of growing interest for physicians and parents alike. In the US, ADHD is the most common behavioral disorder among children and adolescents [1], with the prevalence rising over the last decades. Parent-reported ADHD cases of children ages 4-17 years in the US translated to increased prevalence from 7.8% in 2003 to 11% in 2011 [2] and to 9.5% for 2011-2013 [3]. On a worldwide scale, ADHD prevalence increased from 5.29% in 2007 [4] to 5.9-7.1% in 2012 [5], with the latest meta-analysis estimating it at 7.2% [6]. In Israel, ADHD prevalence, according to the Survey of Mental Health, was estimated at 3% among adolescents in a representative national sample of 14- to 17-year-olds [7]. The increase in ADHD prevalence, incidence, and treatment by medication may partly be a reflection of changing attitudes towards the disorder and its treatment. With the diagnostic process usually involving reports of teachers or parents, the method in which ADHD prevalence is measured could affect the results, depending on how much emphasis is placed on these reports. Methods of measuring prevalence using teacher or parent questionnaires or both, along with a direct interview, can change the estimation of prevalence [4, 8]. Variability in results by geographical region and the DSM (Diagnostic and Statistical Manual of Mental Disorders) edition used were reported recently [6], but an earlier analysis of past studies by Polanczyk et al. [9] has revealed that geographical location and year of study are not necessarily associated with the variability of results; rather the differences were mostly explained by the characteristics of the methodology employed in a study.

According to Getahun et al. [10], relying on parents’ or teacher’s reports to diagnose ADHD in children tends to result in prevalence overestimation. In contrast using stringent diagnostic criteria that included expert physicians and the use of a formal questionnaire in a large health care organization [10], yielded a much lower prevalence (3.1% for 2010). A similarly low prevalence of 2.5% among children of 3-17 years of age was also reported after analyzing data from a large German research database, where the study relied on medical records detailing physician diagnosis and / or medication treatment [11]. It should be emphasized that ADHD diagnosis based on ICD (International Statistical Classification of Diseases and Related Health Problems) 10 and DSM IV by the different studies might contribute to the variability in the observed rates but not to the consistently observed increase of ADHD prevalence.

Information on the ADHD incidence rate is published less frequently, but points to an increase that is similar to the published prevalence data. In a Danish nationwide sample of people aged 4-65 years for the period 1995-2010, the incidence rate increased from 7.3 to 91.2 per 100,000 people [12] while incidence data on diagnosed ADHD from the United Kingdom showed an increase from 6.9 per 100,000 population in 1998 to 12.2 per 100,000 in 2007, and a decrease to 9.9 per 100,000 by 2009 [13].

For some parents, treating ADHD with medication may be the preferred approach, and an increasingly common one. Data from parents’ reports in the US indicates that 69% of children diagnosed with ADHD aged 4-17 years currently receive medication [14]. The prevalence of pharmacologically treated ADHD in the United Kingdom increased between 2003 and 2008 in the age category of 6–12 years, from 0.48% to 0.92%, and from 0.36% to 0.74% for ages 13-17 [15]. In the Netherlands, the prevalence of treated children aged 6-17 years increased between the years 2000-2007 from 1.1% to 2.1% [16]. Prevalence of treated ADHD for all ages in Taiwan increased during 2000–2005 from 0.065% to 0.145% patients [17]. Prevalence of medicated children with ADHD, estimated by using the national records of drug prescription in Israeli children from 6 to 18 years, was 7.5% for the year 2011 [18].

The different methodologies utilized in various studies and nations make the comparison of rates and treatments of ADHD very challenging. Therefore, it is important to use the same inclusion criteria when evaluating ADHD annual trends.

The aim of the present study was to investigate the prevalence, incidence, and pharmacological treatment of ADHD in children and adolescents between 2005 and 2014, in a large cohort, in an attempt to better understand the reasons behind any significant changes in the number of cases of ADHD being diagnosed and pharmacologically treated.

## Methods

### Case identification

We examined data of ADHD diagnosis from the computerized database of the second largest health maintenance organization (HMO) in Israel, Maccabi Healthcare Services, which provides services to 25% of Israel’s 8.4 million citizens.

In Israel, the diagnosis of ADHD and the first recommendation for medication is expected to be given by a neurologist (adult or pediatric) or a psychiatrist (adult or pediatric) and, over the past 7 years, also by qualified pediatricians recognized by the Ministry of Health upon completion of a course on ADHD diagnosis and treatment [19]. The necessary components include the use of the updated DSM criteria and a formal diagnostic questionnaire for parents and teachers.

Using Maccabi’s computerized database, the following key words were used for case identification – Attention Deficit Hyperactivity Disorder (ADHD) Combined Type, ADHD Predominantly Inattentive Type, ADHD Predominantly Hyperactive type, ADHD Not Otherwise Specified.

### Case definition

A major challenge in defining incidence and prevalence is in case definition. A case of ADHD was defined as any child with an ADHD diagnosis aged 5-17 years (17 and 364 days) between the years 2005-2014, with a physician-recorded ADHD diagnosis and / or two purchases of ADHD medication. Children with additional diagnoses, such as autism, were not excluded.

### Case ascertainment

**Fig. 1:**
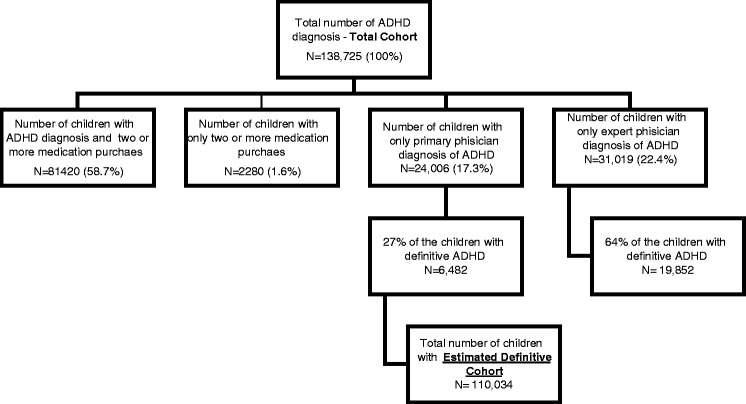
Calculation of the Estimated Total Cohort

Maccabi Healthcare Services’ patient records include those with an ADHD diagnosis recorded by an expert or primary physician, and ADHD medication purchases. Some of the records included only a diagnosis (39.7%), while other records contained both an ADHD diagnosis and medication purchase (58.7%), and a few cases included only the purchase of ADHD medications (1.6%) without noting ADHD in the diagnosis section. All of these records comprised the **total cohort** (Fig. 1).

At this stage, we aimed at estimating the number of children with a definitive ADHD diagnosis. Our basic premise when ascertaining a definitive diagnosis is that ADHD medication is highly targeted, and is therefore unlikely to be prescribed to treat anything other than ADHD. Thus, all children with two or more purchases of ADHD medication (with or without an ADHD diagnosis mentioned in their records) were considered as definitive. Of the cases that included medication purchase without a diagnosis (1.6%), we infer that the majority are a documentation error due to the computerized system not requiring physicians to enter a diagnosis in order to prescribe medication. The number of treated cases by itself does not necessarily reflect the true and full prevalence [20]. Those with only an ADHD diagnosis, without purchase of medications for ADHD treatment, included definitive cases that did not require treatment by medication or those where the parents decided not to treat. Other cases were not definitive, and in some instances, the physician has added remarks such as “in investigation”, “suspected” or “most probably”. In other cases, physicians have not included any remarks, but in the comments section, they have mentioned that the diagnosis was not definitive. Thus, qualifying as a definitive diagnosis requires either two or more purchases of ADHD medication, or a documented ADHD diagnosis without any of the aforementioned added remarks in the comments section. The electronic search tool for the database can identify only the ADHD diagnosis, but not the comments, and therefore all the cases were included in the search results (total cohort).However, we wanted to exclude cases with no definitive diagnosis.

As since it was not practical to review every record that included a diagnosis but no treatment, a random representative sample of 200 patient records, from all years of the study, that contained only a primary physician diagnosis, and 250 records with only an expert diagnosis, were reviewed. The review process included analyzing physicians’ comments and distinguishing between those cases where the physician labeled ADHD as definitive and those which were still in the process of investigation. Of 200 records made by the primary physicians, 54 were found to have a definitive diagnosis (27%), in contrast to 160 out of 250 records (64%) made by an expert that had a definitive diagnosis. Using this percentage, one can extrapolate that from the total of 24,006 children with an ADHD diagnosis given by primary physicians, only 6482 children were designated to have a definitive diagnosis, whereas from a total of 31,019 children with an ADHD diagnosis given by an expert, 19,852 children were designated as definitive ADHD. Hence the total number of children with an **estimated definitive diagnosis**, 110,034, (79.3% of the total cohort) was the sum of the children with two medication purchases (83,700 children), plus 6482 children with a primary physician’s definitive diagnosis and 19,852 children with an expert’s definitive diagnosis (Fig. 1). We assumed that the percentage calculated for the definitive cases would apply equally to all study years and for both males and females. The estimated definitive diagnosis was used as a numerator in the analysis.

### Data analysis

ADHD prevalence was calculated in two ways.

1. For the first prevalence measure, we determined for each year the total number of children aged 5-17 years (17 and 364 days) who had an estimated definitive ADHD diagnosis in the years 2005-2014. We divided that figure by the total number of children in that age group who were registered with Maccabi Healthcare Services for the given year.
2. For the second ADHD prevalence figure, we measured prevalence for each year among three age subgroups: 5-8, 9-12, and 13-17 years.

Subsequently, we calculated the annual estimated definitive diagnosis ADHD incidence rate as the total number of new cases of ADHD diagnosis in children aged 5-17 years (17 and 364 days) in each year from 2005 to 2014, divided by the total membership in that age group for the same year. We also measured the yearly incidence in the three age subgroups (5-8y, 9-12y, and 13-17y).

The prevalence of children who received ADHD medications was calculated by dividing the number of children who received at least two purchases in a specific year by the number of children of the estimated definitive cohort. We also calculated the prevalence of children who received ADHD medication for the years 2005 and 2014 by dividing the number of children treated by the number of all children registered with Maccabi Healthcare Services in those specific years.

### Data on socioeconomic status

We explored whether ADHD prevalence varied by socioeconomic status (SES) based on a social scale that divides geographic locations into different socioeconomic categories on a scale ranging from 1 to 20, where 1 is the lowest SES and 20 is the highest, based on residence area [21]. We assigned each patient a number based on his reported residence, and for the purposes of our analysis, we divided the figures into five groups, where 1-4 is the lowest, 5-8 is low average, 9-12 is average, 13-16 is high average, and 17-20 is high.

### Statistical analysis

A sample size of 250 records from all study years and both genders from the group of children with expert ADHD diagnosis has 80% power to estimate rate of 0.6 of definitive ADHD from this subgroup with 95% Confidence Interval for this rate.

A sample size of 200 records from all study years and both genders with ADHD from the group of children with only primary physician ADHD diagnosis have 80% power to estimate rate of 0.2 of definitive ADHD from this sub-group with 95% Confidence Interval for this rate.

Descriptive statistics of patient data is expressed as numbers and percentages for dichotomous variables. The 99% Confidence Interval for proportions were provided for the rates of prevalence and incidence between years. The Chi-square test for categorical variables was performed to determine significant differences in SES between ADHD diagnosed and all Maccabi healthcare services members.

All analyses were conducted using standard statistical software (SPSS version 22, Inc., Chicago, IL).

## Results

The results presented in this section relate to the estimated definitive ADHD cohort. The ADHD prevalence rate increased from 6.8% to 14.4% (*p* < 0.001) between 2005 and 2014 (Table 1). If we were to take into consideration all mentions of ADHD made by a physician (total cohort) the ADHD prevalence for 2014 would have been even higher, at 18.1%.

**Table 1:** ADHD Prevalence for 5-18 years old children by year and gender

Year	Percentage of male diagnosed EDC (99% CI)	Percentage of female diagnosed EDC (99% CI)	Percentage EDC (99% CI)
2005	9.9 (9.7-10.1)	3.5 (3.4-3.6)	6.8 (6.7-6.9)
2006	11.1	4.2	7.7
2007	12.0	4.8	8.5
2008	13.0	5.6	9.4
2009	14.1	6.3	10.3
2010	15.3	7.3	11.4
2011	16.5	8.3	12.5
2012	17.3	9.1	13.3
2013	18.0	9.9	14.1
2014	*18.3 (18.1-18.5)	10.4* (10.2-10.6)	14.4* (14.3-14.5)

*EDC* Estimated Definitive Cohort, *CI* Confidence Interval

*P < 0.01

While the prevalence among males almost doubled in that time period (9.9% in 2005 to 18.3% in 2014), the female prevalence rate tripled (3.5% to 10.4%) (Table 1).

The male to female ratio decreased from 2.94 in 2005 to 1.86 in 2014 (p < 0.001). The prevalence among boys with ADHD changed dramatically for the group aged 13-17 years (from 11.41% in 2005 to 25.82% in 2014), and lesser (although still statistically significant (*p* < 0.01)) for the youngest group of 5-8 years old (Table 2).

**Table 2:** ADHD Prevalence for different age group, year and gender

Year	Percentage of 5-8 year old -female EDC (99% CI)	Percentage of 5-8 year old male EDC (99% CI)	Percentage of 9-12 year old -female EDC (99% CI)	Percentage of 9-12 year old – male EDC (99% CI)	Percentage of 13-17 year old - female EDC (99% CI)	Percentage of 13-17 year old - male EDC (99% CI)
2005	2.08 (1.9-2.2)	5.74 (5.5-6.0)	4.72 (4.5-4.9)	13.08 (12.7-13.4)	3.95 (3.75-4.15)	11.41 (11.1-11.7)
2006	2.20	5.82	5.49	14.19	5.15	13.72
2007	2.41	5.88	6.18	15.12	6.08	15.42
2008	2.72	5.99	6.81	15.94	7.25	17.45
2009	2.87	6.39	7.70	16.61	8.47	19.53
2010	3.23	7.22	8.59	17.47	10.09	21.11
2011	3.54	7.77	9.62	18.52	11.66	22.76
2012	3.58	7.62	10.74	19.99	13.29	24.75
2013	3.50	7.52	11.08	20.30	14.63	25.15
2014	*3.27 (3.1-3.4)	*6.98 (6.75-7.2)	*11.40 (11.1-11.7)	*20.84 (20.5-21.2)	*15.69 (15.4-16)	*25.82 (25.45-26.1)

*EDC* Estimated Definitive Cohort, *CI* Confidence Interval

*P < 0.01

The female prevalence for the youngest group shows a similarly modest change (*p* < 0.01) from 2005 to 2014, but there was significant change for the group aged 9-12 years (4.72% in 2005 to 11.4% in 2014) and an even more significant increase for the adolescent 13-17 years group (3.95% in 2005 to 15.69% in 2014) (Table 2).

In a similar manner, ADHD incidence also increased over the years, starting from 2005 and reaching a peak in 2011 (23.78 per 1000 children) before declining in 2014 in both sexes (Table 3).

**Table 3:** ADHD Incidence for 5-18 years old children by year and gender

Year	New cases Per 1000 Male – EDC (99% CI)	New cases Per 1000 Female – EDC (99% CI)	Total new cases per 1000 – EDC (99% CI)
2005	20.21 (19.4-21.0)	9.66 (9.1-10.2)	15.07 (14.6-15.6)
2006	20.10	9.96	15.16
2007	20.24	10.53	15.51
2008	22.92	12.84	18.01
2009	25.05	14.12	19.73
2010	27.41	17.03	22.37
2011	28.55	18.73	23.78
2012	26.69	17.90	22.42
2013	26.78	19.19	23.09
2014	*22.95 (22.2-23.7)	*16.12 (15.5-16.8)	*19.63 (19.1-20.1)

*EDC* Estimated Definitive Cohort, *CI* Confidence Interval

*P < 0.01

Overall, the population prevalence of medication usage by estimated definitive diagnosed children and adolescents with ADHD was 3.57% of all children enrolled in Maccabi Healthcare Services in 2005 and 8.51% (*p* < 0.001) in 2014 (for males, the prevalence of medication usage increased from 5.34% in 2005 to 10.9% in 2014, and from 1.71% to 5.98% for females). For 2014, the lowest usage of medication among those with an ADHD definitive diagnosis was seen for males and females in the 13-17 years’ category, at 55.1% and 57.1% respectively, while the highest medication usage was seen in the group aged 9-12 years, at 65.3% and 58.5% respectively (Table 4). Overall, the use of medication increased moderately but with statistical significance between 2005 and 2014, with small difference between males and females.

**Table 4:** Medication purchases by age group, year and gender

Year	Percentage of 5-8 year old females with ADHD MP from EDC (99% CI)	Percentage of 5-8 year old males with ADHD MP from EDC (99% CI)	Percentage of 9-12 year old females with ADHD MP from EDC (99% CI)	Percentage of 9-12 year old males with ADHD MP from EDC (99% CI)	Percentage of 13-17 year old females with ADHD MP from EDC (99% CI)	Percentage of 13-17 year old males with ADHD MP from EDC (99% CI)
2005	43.7 (41.3-47.1)	48.3 (46.3-50.3)	49.0 (46.6-51.4)	58.3 (56.9-59.7)	51.0 (48.4-53.6)	52.5 (51.0-54.0)
2006	42.5	47.6	48.6	56.9	50.5	50.7
2007	45.1	48.1	48.6	56.5	47.9	49.5
2008	43.6	46.7	49.0	56.4	50.7	50.4
2009	43.8	49.0	51.7	57.5	51.8	52.0
2010	49.1	53.1	54.8	60.3	54.7	53.8
2011	51.9	56.4	57.3	63.4	57.5	55.1
2012	51.8	58.3	57.4	64.2	56.5	55.1
2013	56.3	62.1	59.7	65.5	58.5	55.9
2014	*57.8 (55.2-60.4)	*63.3 (61.6-65.0)	*58.5 (57.1-59.9)	*65.3 (64.3-66.3)	*57.1 (56.0-58.2)	*55.1 (54.3-55.9)

*EDC* Estimated Definitive Cohort, *MP* Medication Purchases, *CI* Confidence Interval

*P < 0.01

ADHD diagnosis was less frequent among the lower SES and more frequent among the average and high average SES (Table 5).

**Table 5:** Percentage Distribution of Socioeconomic Status among all Children and with Definitive ADHD in 2014

SES Levels	1-4 (Low)	5-8 (Low Average)	9-12 (Average)	13-16 (High Average	17-20 (High)
ADHD Diagnosed *N* = 69,041*	5.2**	18.7**	30**	28.1**	17.9
All Maccabi *N* = 484,286	7.3	21.8	27.3	25.5	18.1

*SES* Socioeconomic Status, *Maccabi* Maccabi Healthcare Services

*Fraction of the Total Cohort that Socioeconomic Status (SES) could be calculated

** *P* < 0.01 between ADHD Diagnosed and All Maccabi patients

## Discussion

This discussion will focus on challenges in understanding the recent increase in ADHD prevalence. We identified a prevalence rate which is double that of the worldwide prevalence published recently [6] and also higher than the 11% prevalence calculated from the number of parents who reported that their children had received an ADHD diagnosis by a physician [2]. In our study, the prevalence was higher for males, especially for the age group of 13-17 years, where a quarter had an ADHD diagnosis. However, the female prevalence, although still lower than males, has tripled during the last 10 years, and the proportion of females with ADHD has increased. A similar trend was recently reported by Collins and Cleary [22].

The increase in prevalence is dramatic, and while it is still too early to determine the exact causes for it, there are several different factors that should be discussed as contributing to the upward shift.

### Physician challenges in diagnosing ADHD

Presently, ADHD still does not have biological markers for diagnosis and hence the diagnosis relies mostly on physicians’ education and practice [23]. In Israel, the Ministry of Health regulates the process of ADHD diagnosis and the recommended medications. While neurologists, psychiatrists and trained pediatricians are expected to adhere to the American Academy of Pediatrics Guidelines for ADHD diagnosis [1], it is difficult to examine how closely they follow these recommendations. In comparison, the diagnosis of the autistic spectrum requires the DSM IV [24] (or DSM 5 [25]) criteria to be fulfilled and documented in order for the patient to be eligible to receive government support [26]. In the case of ADHD, the lack of DSM documented criteria means that diagnosis can be made more easily, thus potentially skewing the prevalence rate. There is always the possibility that ADHD medication is being prescribed to children who in reality do not fulfill ADHD criteria [27]. On the other hand, since the evaluation process has not changed during the years of our research, this putative cause is less likely to explain the sharp rise in prevalence. A change in DSM edition was also argued by some investigators as a cause for the increased prevalence [28]. During the study years, physicians used the DSM IV criteria, and the influence of the new DSM 5 might have even lowered the rate of new diagnosis during 2014.

### Parental challenges during the process of evaluation for ADHD

The pool of Israeli physicians qualified to make ADHD diagnoses has grown since 2007, when pediatricians have been permitted to evaluate only upon completing a Ministry-approved course on diagnosing ADHD. The increased number of physicians who are able to provide a diagnosis has made ADHD clinics more approachable for parents around the country and this could have influenced the ease of obtaining an evaluation. Social stigma could also play a role in parents’ decision to seek help [29]. For example, approaching a trained pediatrician might seem easier for some parents than getting the same diagnosis from a psychiatrist. The attitude of parents towards ADHD has also changed over the years, and in our clinical experience, more parents appear to consider ADHD diagnosis and treatment as a means to improve their child’s achievements (especially if they are underperforming academically), commonly with the aid of medications. The children themselves often state during the evaluation that they want ADHD medications “like their friends”, and some parents seek multiple evaluations when an ADHD diagnosis has been excluded by one professional [30]. Expanding on the idea of ADHD medications being used as a means to enhance performance, the former chairman of the Ethic Committee of the Israeli Medical Association has alluded to the possibility that off-label medication might be used by those who seek cognitive enhancement without being diagnosed with a disorder [31]. These new parental considerations, which lead to increased testing and diagnosis for their children, are equally relevant to females, and may thus contribute to the dramatic observed increase in the prevalence of female ADHD diagnosis.

Some studies reported of trends towards lower SES among children diagnosed with ADHD, while others have not shown it [5]. Our data suggests that ADHD was diagnosed more commonly in the average and high average SES strata than expected when comparing it to the total population. If one relates this to the previous paragraph, one may be able to associate higher SES households with the aforementioned parental considerations regarding academic success.

We believe that the above reasons make the possibility of over - diagnosis [29] something to be taken into consideration.

### The challenges of changed environment

Other factors, such as environmental, have been suggested in explaining the increase in ADHD prevalence [32]. The attention span of children and adolescents might be negatively impacted by television viewing and video games [33, 34], as well as poor sleep patterns related to excessive electronic media using habits [35]. There is a massive increase in “screen time” with the use of smartphones and we believe, based on clinical experience, that this change could play a role in the increased rate of ADHD, as reported by Zheng et al. [36]. Furthermore, the “equalizing” nature of environmental influences may contribute to the understanding of the increase in female ADHD diagnosis prevalence.

In parallel to prevalence, during the years of this study, the incidence rate has also increased, especially among females, although a decrease in incidence was noted in 2014. It could be argued that the war that took place in Israel during the summer of 2014 might have influenced parents’ decision whether to seek help for ADHD in their children since they pursue medical aid only for more urgent concerns. As this is still relatively new data, we will continue to follow the incidence over the next few years to verify whether there has been a real decline of newly occurring cases of ADHD among Israeli children and adolescents.

Our findings have corroborated a dramatic rise in the prevalence of Israeli children being treated with medications for ADHD. This translates to more than one in every ten males aged 5-17 years treated while the prevalence of treated females increased even more dramatically. This prevalence figure is higher than the 6.1% reported in the US [14] as well as the prevalence reported in the Netherlands and the United Kingdom [15, 16]. These differences in prevalence of medication treatment across countries could reflect differences in approach of caregivers and physicians for treating ADHD using medication.

### Strengths and limitations

The current study is based on physician-recorded ADHD diagnoses, which contains nationwide population data, and does not rely on insurance claims or parents’ reports that could be biased. Our data is generated from clinicians’ evaluation, making it the most reliable source of information available. In addition, by calculating both a total cohort and an estimated definitive cohort, we have been able to increase specificity by looking at the prevalence of the children for whom ADHD was considered by an expert physician.

However, a potential limitation has been in calculating the **estimated definitive prevalence** using only a representative sample of medical records. With Maccabi Healthcare Services being the second largest HMO in Israel, it is impractical to go through every single electronic medical record on its digital database that included a potential ADHD diagnosis. We have assumed that the selected random sample is representative, and yields a true picture of all study years. We further acknowledge that by utilizing extrapolation techniques in certain cases, it is not possible to discern which individual level variables (e.g., SES) are responsible for the significant changes in the number of cases of ADHD being diagnosed and pharmacologically treated.

## Conclusions

The large increase seen in the prevalence, incidence and drug therapy for ADHD diagnoses, highlight challenges in distinguishing between methods of collection and ascertainment of children with the condition, versus the possibilities of genuine, true increase rates of ADHD. However, while we acknowledge that over-diagnosis exists, one has to bear in mind that from that moment on, the children live with the diagnosis, along with their self and peers’ perception of it, and often with medication.

We suggest stricter adherence to the diagnostic criteria. In addition, we suggest that physicians rigorously document the fulfilled criteria, as well as explain the functional ramifications they impose on the child, especially prior to prescribing medication.

## Abbreviations

ADHD: Attention Deficit Hyperactivity Disorder
DSM: Diagnostic and Statistical Manual of Mental Disorders
HMO: Health Maintenance Organization
ICD: International Statistical Classification of Diseases and Related Health Problems
SES: Socioeconomic status
